# Effects of Heterogeneity on Cancer: A Game Theory Perspective

**DOI:** 10.1007/s11538-023-01178-9

**Published:** 2023-06-19

**Authors:** Annick Laruelle, André Rocha, Claudia Manini, José I. López, Elena Inarra

**Affiliations:** 1 Department of Economic Analysis (ANEKO), University of the Basque Country (UPV/EHU), Avenida Lehendakari Aguirre, 83, 48015 Bilbao, Spain; 2 IKERBASQUE, Basque Foundation of Science, 48011 Bilbao, Spain; 3 Department of Industrial Engineering, Pontifical Catholic University of Rio de Janeiro, Rua Marquês de São Vicente 225, Gávea, Rio de Janeiro, RJ CEP 22451-900 Brazil; 4 Department of Pathology, San Giovanni Bosco Hospital, 10154 Turin, Italy; 5 Department of Sciences of Public Health and Pediatrics, University of Turin, 10124 Turin, Italy; 6 Department of Pathology, Cruces University Hospital, 48903 Barakaldo, Spain; 7 Biomarkers in Cancer Group, Biocruces-Bizkaia Research Institute, 48903 Barakaldo, Spain; 8 Institute of Public Economics, University of the Basque Country (UPV/EHU), Avenida Lehendakari Aguirre, 83, 48015 Bilbao, Spain

**Keywords:** Cancer, Intratumor heterogeneity, Hawk–dove game, Evolutionarily stable strategy

## Abstract

In this study, we explore interactions between cancer cells by using the hawk–dove game. We analyze the heterogeneity of tumors by considering games with populations composed of 2 or 3 types of cell. We determine what strategies are evolutionarily stable in the 2-type and 3-type population games and what the corresponding expected payoffs are. Our results show that the payoff of the best-off cell in the 2-type population game is higher than that of the best-off cell in the 3-type population game. When these mathematical findings are transferred to the field of oncology they suggest that a tumor with low intratumor heterogeneity pursues a more aggressive course than one with high intratumor heterogeneity. Some histological and genomic data on clear cell renal cell carcinomas is consistent with these results. We underline the importance of identifying intratumor heterogeneity in routine practice and suggest that therapeutic strategies that preserve heterogeneity may be promising as they may slow down cancer growth.

## Introduction

Precision oncology loses much of its efficiency when significant genetic differences appear between different regions of the same tumor and remain hidden because of incomplete sampling. In terms of therapy this means that some areas of the tumor will respond to treatment while others will not. The reliable identification of such intratumor heterogeneity (ITH) so as to assure the efficacy of current therapies is a cornerstone of modern oncology (Middleton et al. 2021). From a genomic perspective, temporal and spatial heterogeneity develop in every tumor, following different evolutionary models. To date, four different patterns have been identified: Linear, branching, neutral, and punctuated (Davis et al. 2017). Branching and punctuated patterns have been extensively analyzed in clear cell renal cell carcinomas (CCRCC), an aggressive variant of renal cancer (Turajlic et al. 2018b).

It is widely agreed that the punctuated pattern, corresponding to tumors that display low ITH, shows a worse prognosis than the branching pattern, which corresponds to high ITH. Motivated by this finding, we set out to design a game theoretical model that represents it well in oncology and to see whether the solution to that model is consistent with it.

Game theory has recently been identified by 33 expert oncologists as a key model for understanding tumorigenesis and potentially guiding therapy (Dujon et al. 2021). Several games (or a combination of games) have been used to study cancer, including the prisoner’s dilemma (West et al. 2016), the hawk–dove game (Tomlinson 1997; McEvoy 2009; Kareva and Karev 2019; Swierniak et al. 2019, 2020), coordination games (Bayer et al. 2022) and public good games (Nogales and Zazo 2021).

In particular, evolutionary game theory is a powerful tool for studying cancer as it models how types of cells compete with each other for resource and space, and how their strategies evolve over time. See for instance, Wölfl et al. (2022) for a review of this literature. An evolutionarily stable strategy (ESS) guarantees that no mutant strategy can invade the population (Maynard-Smith and Price 1973; Maynard-Smith and Parker 1976; Maynard-Smith 1982). That is, an ESS is a strategy which, when adopted by a population of individuals, cannot be invaded by any alternative strategy.^1^ In the context of cancer, ESS can be used to understand the evolution of tumor cell populations.

In this paper we use a non cooperative game and the ESS as a solution concept. This approach has several advantages. From a mathematical perspective, it avoids having to explain possible evolutionary trajectories that might conflict with empirical oncological findings. From a medical perspective, it is not feasible to conduct a large number of biopsies over time in patients: The evolutionary trajectories of cells over time cannot be assessed in the clinical practice due to deontological reasons. From a game theory perspective, the ESS can be applied in games in normal form - the archetypal model of interactions.

Our contribution consists of developing a game theoretical model that relates ITH to the prognosis of the tumor. More specifically, we start by choosing the hawk–dove game because of its simplicity and its affinity to cell behavior.^2^ Hawk–dove games are defined by two parameters: The resource (v) and the cost (c). The resource in tumors consists of a varied spectrum of diffusible factors released into the medium by tumor and/or non-tumor cells. An example is the fibroblast activation protein (FAP) produced and released by a specific sub-type of cancer-associated fibroblasts (Errarte et al. 2020). The cost is the energy required to obtain the resource. The Atkinson level enables the energy spent by any cell, normal or neoplastic, to be measured. It takes into account the relative cytoplasmic concentrations of adenosine tri-(ATP), di-(ADP), and mono-(AMP) phosphate (the essential molecules fueling all cellular processes) (De la Fuente et al. 2014). The hawk–dove game is applied under the assumption that cells do not recognize their own type but detect the type of their opponents. Examples of how cancer cells behave differently depending on the recognition of their respective cellular contexts are available in the literature (Maruyama and Fujita 2017).

Then we use a heterogeneous game (Inarra and Laruelle 2012), i.e. we consider heterogeneous populations composed of two or three types of cells: The 2-type population model represents low ITH (punctuated pattern) and the 3-type population model represents high ITH (branching pattern). Clinical practice suggests that encountering more than three different types of cells in a tumor is quite unusual. Therefore, we do not consider populations composed of four or more types.

Finally, we compare the ESS for the 2-type and 3-type populations models. In the ESS, different types of cells obtain different levels of payoffs. The cells that obtain the highest expected payoffs are the fittest ones, i.e. those that divide at the highest rate, increasing their proportion over time. Thus, these cells determine how aggressive the tumor is. Therefore, we compare the expected payoffs of the fittest cells for the two population games to assess the prognosis of tumors. The findings obtained are consistent with what is observed in histological and genomic studies on CCRCC (Turajlic et al. 2018a, b; Manini et al. 2022).

The paper is organized as follows. Section 2 presents a case study that enables us to develop our 2-type and 3-type game theoretical models. Section 3 sets out the game theoretical models and gives the main results. Section 4 contains a discussion and our conclusions.

## A Case Study

In clinical practice, tumors are classified using two types of approaches: Histological and genomic. In the former, tumor classification is based on the morphological features of tumor cells under the microscope when pathologists assign tumor grading following internationally accepted criteria. Most tumors in the body are graded from 1 to 3, or more rarely from 1 to 4, where grade 1 means the lowest aggressiveness and grade 3 (or 4) means the highest aggressiveness. In such analyses, a distinction can also be drawn between low and high ITH.

In genomic analysis, a distinction can be drawn between punctuated and branching cancer evolutionary patterns as discussed in Davis et al. (2017). With the recent development of next-generation sequencing (NGS)-based platforms, it has become feasible to conduct concurrent analysis on hundreds of genes or even the entire genome using small quantities of tumor tissue collected by needle biopsy.

The two types of tumor analysis agree that low ITH (corresponding to the punctuated evolutionary pattern) is more aggressive than high ITH ( corresponding to the branching pattern) (Manini et al. 2022; Turajlic et al. 2018b).

To study the effects of low and high ITH in CCRCC, Manini et al. Manini et al. (2022) have recently reviewed a series of 28 exhaustively sampled CCRCC focusing specifically on the variability of tumor grades. The follow-up on all patients varied between 5 and 10 years. More than 1500 tumor samples were assessed, averaging more than 50 samples per case. In 5 of the 28 cases a single grade was observed in all the samples analyzed. In 15 cases two different grades were observed; and in 8 cases three grades were observed. Cases with two different grades across all the regions analyzed were considered to show low ITH, whereas cases with three different grades were considered as high ITH.

**Table 1:** Cases of CCRCC showing some degree of ITH, adapted from Manini et al. (2022)

Case	Proportion of the Highest Grade (%)	Heterogeneity (ITH)	Outcome
1	10	Low	AWD
2	5	High	AwoD
3	80	Low	AwoD
5	60	Low	AWD
7	5	High	AwoD
8	10	High	AwoD
9	70	Low	DOD
10	90	Low	DOD
11	90	Low	DOD
12	70	Low	DOD
13	80	Low	DOD
14	70	Low	DOD
15	5	High	AwoD
16	10	Low	AWD
17	5	High	AwoD
18	50	Low	DOD
19	90	Low	AWD
20	20	High	AwoD
21	5	High	AwoD
22	90	Low	AWD
23	70	Low	DOD
24	90	Low	DOD
25	10	High	AwoD

Outcome at last contact—AWD, alive with disease; AwoD, alive without disease; DOD, died of disease

The 23 cases that display some heterogeneity are summarized in Table 1.^3^ For each case we give the level of heterogeneity, the percentage of the highest grade and the outcome at last contact. It can be observed that 9 of the 15 patients with tumors showing low ITH died of the disease, 5 were alive with the disease, and 1 was alive without the disease at last contact. By contrast, all 8 patients with tumors showing high ITH were alive without the disease at last contact. Moreover, with the exception of Cases 1 and 16, all tumors showing low ITH also show high proportions of the highest grade cells.

The prognosis of patients with low ITH and high proportions of the most malignant type of cells is bad. Indeed all but two of them died of the disease. Conversely, all the patients with tumors showing high ITH are alive and show small proportions of the highest grade cells.

## The Hawk–Dove Game in Tumors

In this section we present our game theoretical approach based on the case study in the previous section. We first introduce the hawk–dove game in a homogenous population. To adapt our modeling to the ITH problem we refer to cells rather than players, and seek to justify every assumption that we make in the cancer setting. Then we introduce the hawk–dove game in populations with 2 different types of cells (referred to as 2-type populations) and 3 different types of cells (referred to as 3-type populations) exemplifying interactions within cells in low ITH and high ITH tumors, respectively.

### Homogenous Population

We consider a tumor formed by a population of *n* cells. Encounters between cells are bilateral and in each encounter a cell can behave aggressively, like a hawk, or passively, like a dove, to acquire a resource *v*. If one cell is aggressive and its opponent is passive, the former obtains the resource and the latter gets nothing. If both cells are aggressive there is a fight and the winner gets the resource while the loser bears a cost $c>v$. Assuming that they both have the same probability of winning, the expected reward, (“expected payoff” hereafter) for each cell is $(v-c)/2$. If both cells are passive, one withdraws and gets nothing while the other takes the resource. Assuming that they both have the same probability of withdrawing, the expected payoff for each cell is *v*/2. These contingencies are summarized in the following payoff matrix.

$$\begin{aligned} \begin{array}{c|cc} &{} \hbox {hawk} &{} \hbox {dove} \\ \hline \hbox {hawk} &{} \frac{v-c}{2} &{} v \\ \hbox {dove} &{} 0 &{} \frac{v}{2} \end{array} \end{aligned}$$

A strategy denoted by $\alpha$ represents the probability of a cell being aggressive on meeting another cell. A cell can choose to play hawk ($\alpha =1$), dove ($\alpha =0$) or a mixed strategy ($0<\alpha <1$). The expected payoff of a cell that plays $\alpha$ when its opponent plays $\beta$ is given by $u(\alpha ,\beta )$:

1 $$\begin{aligned} u(\alpha ,\beta )=\frac{v}{2}(1-\beta )+\frac{c}{2}\left( \frac{v}{c}-\beta \right) \alpha . \end{aligned}$$

The solution concept applied to solve this game is the evolutionarily stable strategy (ESS). An ESS is a strategy that maximizes the expected payoff of a player when its opponent chooses the same strategy, i.e. a symmetric Nash equilibrium. An ESS also guarantees that no mutant strategy can invade the population. It is known that strategy *v*/*c* is the only ESS (Maynard-Smith 1982) when the population is homogeneous, i.e. composed of a single type of cell.

### Heterogeneous Population

To analyze the effect of ITH on the fitness of tumors, we need to compare cells with identical capacities in terms of both the level of resources to which they are exposed and their energy cost in fighting for those resources. Notice that a priori types do not confer any advantage: The same payoff matrix is played in every encounter.

Tumors are usually composed of different types of cells. In our modeling we follow the above case study, and consider two levels of ITH: Low and high. For notation purposes, we define A-cells as the best-off cells, i.e. those with the strictly largest expected payoff, thus those that reproduce fastest and define the aggressiveness of a cancer tumor. A tumor with low ITH is composed of two types of cells, say *A*-cells and *B*-cells. A tumor with high ITH is composed of three types of cells, say *A*-cells, *B*-cells and *E*-cells.

As mentioned in the Introduction, cells are sensitive to their environment and recognize the type of their opponent, but not their own type. Hence, a cell sees itself as *I*-cell, i.e. it does not know its own type, which may be *A*-cell or *B*-cell in a 2-type population game or *A*-cell, *B*-cell, or *E*-cell in a 3-type population game.

#### 2-Type Population Game

Consider a heterogeneous tumor formed by *A*-cells and *B*-cells. A 2-type population game is denoted by $\Gamma _{2}(v,c,x_{A})$, as it can be defined by parameters *v*, *c*, and the proportion of *A*-cells, $x_{A}$. Cells cannot adopt a different strategy according to their own type but are able to choose a different probability of playing aggressively when facing any type of opponent.^4^ Thus, a strategy of a player is a pair ($\alpha _{A},\alpha _{B}$), where $\alpha _{I}$ denotes the probability of behaving aggressively on meeting an *I*-cell ($I=A,B$). In other words, $\alpha _{I}$ indicates the level of aggression received by an *I*-cell. Game $\Gamma _{2}(v,c,x_{A})$ is analyzed in Inarra and Laruelle (2012). It is shown that no strategy with $\alpha _{A}=\alpha _{B}$ is evolutionarily stable. The game has two evolutionarily stable strategies in which cells of one type receive less aggression and obtain a larger expected payoff than cells of the other type.^5^

We focus on the ESS where the *A*-cells have the largest expected payoff and face the least aggression. This ESS depends on the proportion of *A*-cells. We denote it by $$\left( \alpha _{A}^{**},\alpha _{B}^{**}\right) _{\text {ESS}}$$:

$$\begin{aligned} \left( \alpha _{A}^{**},\alpha _{B}^{**}\right) _{\text {ESS} }=\left\{ \begin{array}{ll} \left( 0,\tfrac{n-1}{n-nx_{A}-1}\tfrac{v}{c}\right) &{} \text {if }x_{A}<{\bar{x}} _{A} \\ (0,1) &{} \text {if }{\bar{x}}_{A}<x_{A}<{\bar{x}}_{A}+\frac{1}{n} \\ \left( \frac{(n-1)v/c-n+nx_{A}}{nx_{A}-1},1\right) &{} \text {if }x_{A}>{\bar{x}} _{A}+\frac{1}{n}, \end{array} \right. \end{aligned}$$

where $${\bar{x}}_{A}=\left( 1-\frac{v}{c}\right) \left( 1-\frac{1}{n}\right) $$.

In the ESS the best-off *A*-cells receive less aggression than *B*-cells ($$ \alpha _{A}<\alpha _{B}$$) do. The level of aggression toward the latter increases when the proportion of *A*-cells increases, until full aggression ( $$\alpha _{B}=1$$) is reached (for $$x_{A}>{\bar{x}}_{A}$$). Also, if the proportion of *A*-cells is below a threshold ($$x_{A}<{\bar{x}}_{A}+1/n$$), *A* -cells suffer no aggression ($$\alpha _{A}=0$$) and aggression is concentrated only on *B*-cells. Above that threshold, however, *A*-cells do receive some aggression. Since only the expected payoff of the best-off cells is of interest in this context, we focus our analysis on the expected payoff of those cells in the ESS, denoted by $$U_{A}^{**}(x_{A})$$, which is given by:

2 $$\begin{aligned} U_{A}^{**}(x_{A})=\left\{ \begin{array}{ll} \tfrac{v}{2}(1-\tfrac{v}{c})+\tfrac{v^{2}}{2c}\tfrac{2n(1-x_{A})-1}{ n-nx_{A}-1} &{} \text {if }x_{A}<{\bar{x}}_{A} \\ \tfrac{v}{2}(1-\tfrac{v}{c})+\tfrac{v}{2c}\tfrac{v(n-1)+cn(1-x_{A})}{n-1} &{} \text {if }{\bar{x}}_{A}\le x_{A}\le {\bar{x}}_{A}+\frac{1}{n} \\ \tfrac{v}{2}(1-\tfrac{v}{c})+\tfrac{c-v}{c}\tfrac{vn(1-x_{A})}{nx_{A}-1} &{} \text {if }x_{A}>{\bar{x}}_{A}+\frac{1}{n}. \end{array} \right. \end{aligned}$$

Observe that if the proportion of *A*-cells is below a threshold ($x_{A}< {\bar{x}}_{A}$), in the ESS the larger $x_{A}$ is, the larger the expected payoff of the *A*-cells is (see “Appendix A”). Indeed, *A*-cells receive no aggression while *B*-cells receive more aggression as $x_{A}$ increases. In consequence, an *A*-cell more frequently obtains resource *v*, thus increasing its expected payoff. The expected payoff of an *A*-cell peaks when $x_{A}={\bar{x}}_{A}$ and starts decreasing thereafter. This finding suggests that *A*-cells start fighting among themselves for resource *v*.

**Fig. 1:**
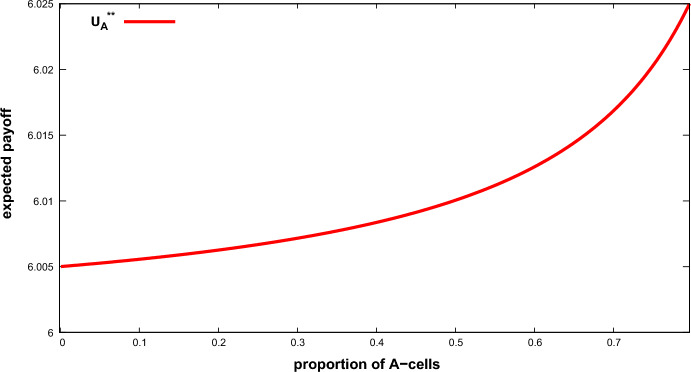
Expected payoff of the best-off *A*-cells in the ESS in a 2-type population game ($$U_{A}^{**}$$) as a function of $$x_{A}$$. Parameters: $$v=10$$, $$c=50$$, $$n=201$$ (Color figure online)

Figure 1*plots the expected payoff of the A-cells as a function of *$$x_{A}$$*in the ESS for *$$x_{A}<{\bar{x}}_{A}$$. This figure shows that both the first and second derivatives are positive: The larger $$x_{A}$$ is, the larger $$U_A^{**}(x_A)$$ is and the larger the increase in $$U_{A}^{**}(x_A)$$ is.

#### 3-Type Population Game

Consider a heterogeneous tumor formed by *A*-cells (in a proportion of $x_{A}$), *E*-cells (in a proportion of $x_{E}$) and *B*-cells (in a proportion of $x_{B}=1-x_{A}-x_{E}$). A 3-type population game is denoted by $\Gamma _{3}(v,c,x_{A},x_{E})$, as defined by parameters *v*, *c*, and the proportions of *A*-cells and *E*-cells.

A strategy is denoted by $$(\alpha _{A},\alpha _{B},\alpha _{E})$$ where $$ \alpha _{I}$$ is the probability of behaving aggressively when facing an *I* -cell ($$I=A,B,E$$). If an *I*-cell plays $$(\alpha _{A},\alpha _{B},\alpha _{E})$$ against an opponent playing $$(\beta _{A},\beta _{B},\beta _{E})$$ its expected payoff is the sum of the probability of meeting a cell of its own type, i.e. $$(nx_{I}-1)/(n-1)$$, multiplied by $$u(\alpha _{I},\beta _{I})$$, and the probability of meeting a cell of each other cell type, i.e. $$ (nx_{J})/(n-1)$$, multiplied by $$u(\alpha _{J},\beta _{I})$$. The expected payoff of an *I*-cell denoted by $$U_{I}$$, gives the following expressions:

3 $$\begin{aligned} U_{A}= & {} \frac{nx_{A}-1}{n-1}u(\alpha _{A},\beta _{A})+\frac{n(1-x_{A}-x_{E}) }{n-1}u(\alpha _{B},\beta _{A})+\frac{nx_{E}}{n-1}u(\alpha _{E},\beta _{A}), \nonumber \\ U_{B}= & {} \frac{nx_{A}}{n-1}u(\alpha _{A},\beta _{B})+\frac{n(1-x_{A}-x_{E})-1 }{n-1}u(\alpha _{B},\beta _{B})+\frac{nx_{E}}{n-1}u(\alpha _{E},\beta _{B}), \nonumber \\ U_{E}= & {} \frac{nx_{A}}{n-1}u(\alpha _{A},\beta _{E})+\frac{n(1-x_{A}-x_{E})}{ n-1}u(\alpha _{B},\beta _{E})+\frac{nx_{E}-1}{n-1}u(\alpha _{E},\beta _{E}). \end{aligned}$$

Moreover, as a cell does not know its own type, what can be maximized is the expected payoff of a generic cell, denoted by *U*. This is computed as the sum of the probability of being *I* multiplied by the expected payoff of an *I*-cell with $I\in \{{A,B,E\}}$:

4 $$\begin{aligned} U=x_{A}U_{A}+(1-x_{A}-x_{E})U_{B}+x_{E}U_{E}. \end{aligned}$$

Substituting (1) into (3) and then substituting (3) into (4), the expected payoff of a generic cell can be written as follows:

5 $$\begin{aligned} U=f(\beta _{A},\beta _{B},\beta _{E})+\sum _{I\in \left\{ A,B,E\right\} }f_{I}(\beta _{A},\beta _{B},\beta _{E})\alpha _{I}, \end{aligned}$$

$$\begin{aligned}{} & {} \text {where }f(\beta _{A},\beta _{B},\beta _{E}) = \frac{v}{2}\left[ 1-\sum _{I}x_{I}\beta _{I}\right] \\{} & {} \quad f_{I}(\beta _{A},\beta _{B},\beta _{E}) = \frac{c}{2}\frac{nx_{I}}{n-1} \left[ \frac{v}{c}\left( 1-\frac{1}{n}\right) -\sum _{I}x_{I}\beta _{I}+\frac{ \beta _{I}}{n}\right] \text { for }I=\left\{ A,B,E\right\} . \end{aligned}$$

As in the 2-type game, whenever cells choose the same strategy the cell that receives the largest payoff is the cell that receives the least aggression. That is, $U_{I}>U_{J}$ is equivalent to $\alpha _{I}<\alpha _{J}$ (for $I,J\in \{{A,B,E\}}$ and$\ I\ne J$). See “Appendix B” for the proof.

We focus on the ESS where the three different types receive different expected payoffs. To make the comparison with the 2-type game, we set $$ U_{A}>U_{B}>U_{E}$$. The following proposition, proven in “Appendix C”, indicates that the ESS depends on the proportions of the *A*-cells and *E* -cells. We denote it by $$\left( \alpha _{A}^{***},\alpha _{B}^{***},\alpha _{E}^{***}\right) _{\text {ESS}}.$$

##### Proposition 1

Let $\Gamma _{3}(v,c,x_{A},x_{E})$ be a 3-type population game. There is an ESS with $U_{A}>U_{B}>U_{E}$ if and only if $x_{A}<{\bar{x}}_{A}$ and $x_{E}<{\bar{x}}_{E}$, where ${\bar{x}}_{E}=\frac{v}{c} \left( 1-\frac{1}{n}\right)$ which is given by:

6 $$\begin{aligned} \left( \alpha _{A}^{***},\alpha _{B}^{***},\alpha _{E}^{***}\right) _{\text {ESS}}=\left( 0,\frac{(n-1)v/c-nx_{E}}{ n-nx_{A}-nx_{E}-1},1\right) . \end{aligned}$$

**Fig. 2:**
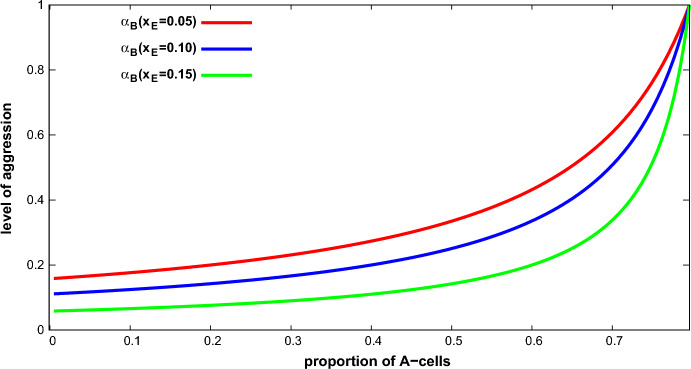
Aggression received in the ESS by *B*-cells ($\alpha _{B}$) in a 3-type population game as a function of $x_{A}$ for different values of $x_{E}$. Parameters: $v=10$, $c=50$, $n=201$ (Color figure online)

As expected, *A*-cells receive less aggression than *B*-cells, which in turn receive less aggression than *E*-cells ($\alpha _{A}<\alpha _{B}<\alpha _{E}$ ). The best-off cells receive no aggression, *B*-cells receive some aggression and *E*-cells receive full aggression. Figure 2 plots the aggression suffered by *B*-cells in the ESS for different proportions of *A*-cells and *E*-cells, showing that: (i) The larger $x_{A}$ is, the greater the aggression suffered by *B*-cells is; and (ii) the larger $x_{E}$ is, the lesser the aggression suffered by *B*-cells is (see “Appendix D”).

Recall that the expected payoff of the best-off *A*-cells is of interest in this context, so we present the expected payoff in the ESS of these cells, denoted by $$U_{A}^{***}(x_{A},x_{E})$$, which is given by:

7 $$\begin{aligned}{} & {} U_{A}^{***}(x_{A},x_{E}) \nonumber \\{} & {} \quad =\frac{v}{2}\left( 1-\frac{v}{c}\right) + \frac{v^{2}}{2c}\frac{2n(1-x_{A}-x_{E})-1}{n-nx_{A}-nx_{E}-1}-\frac{v}{2} \frac{nx_{E}}{\left( n-nx_{A}-nx_{E}-1\right) \left( n-1\right) }. \end{aligned}$$

Observe that the larger $x_{A}$ is, the larger the expected payoff of each *A*-cell is (see “Appendix E”). Indeed, aggression towards *B*-cells increases while *A*-cells receive no aggression (and *E*-cells receive full aggression). As a result, an *A*-cell obtains resource *v* more often and its expected payoff therefore increases. By contrast, the larger $x_{E}$ is, the smaller the expected payoff of each *A*-cell is. As $x_{E}$ increases two opposite effects come into play: On the one hand there are more *E* -cells, which receive full aggression, so overall aggression levels increase, which is beneficial for *A*-cells. On the other hand, as $x_{E}$ increases the aggression received by *B*-cells decreases, which reduces overall aggression, which is detrimental for *A*-cells. This negative effect turns out to outweigh the positive effect, so the expected payoff of each *A* -cell decreases with $x_{E}$.

An important point to highlight here is that there is no ESS with $$ U_{A}>U_{B}>U_{E}$$ when the proportion of *A*-cells or *E*-cells exceeds thresholds $${\bar{x}}_{A}$$ or $${\bar{x}}_{E}$$. However, having $$x_{A}>{\bar{x}} _{A} $$ or $$x_{E}>{\bar{x}}_{E}$$ does not rule out the existence of ESS. In these cases two cell types receive the same expected payoff: Either $$ U_{A}>U_{B}=U_{E}$$ or $$U_{A}=U_{B}>U_{E}$$, reproducing the outcomes of the 2-type population game described above. This can be illustrated as follows for the case $$U_{A}>U_{B}=U_{E}$$.

##### Proposition 2

Let $\Gamma _{3}(v,c,x_{A},x_{E})$ be a 3-type population game. There exists an ESS with $U_{A}>U_{B}=U_{E}$ for $x_{A}> {\bar{x}}_{A}$ which is given by

$$\begin{aligned} \left( \alpha _A,\alpha _B,\alpha _E\right) _{\text {ESS}}=\left\{ \begin{array}{ll} (0,1,1) &{} \text {if }{\bar{x}}_{A}<x_{A}<{\bar{x}}_{A}+1/n \\ \left( \frac{(n-1)v/c-n(1-x_{A})}{nx_{A}-1},1,1\right) &{} \text {if }x_{A}> {\bar{x}}_{A}+1/n. \end{array} \right. \end{aligned}$$

In this ESS, the *E*-cells are not distinguished from the *B*-cells ($\alpha _{B}=\alpha _{E}$). The level of aggression received and the expected payoffs are identical to those obtained in the ESS in the 2-type population game. This is equivalent to a high ITH tumor evolving into a low ITH tumor whenever the proportion of the best-off cells in the tumor becomes large enough. In Table 1 every case in which the proportion of the highest grade is large shows a tumor with low ITH, except those of the patients described in Cases 1 and 16.

### Comparison of Heterogeneous Tumors

As mentioned, the main question here concerns the comparison of the expected payoff of the best-off *A*-cells in the 2-type and 3-type population games. Clearly, the comparison is pertinent for equal proportions of *A*-cells in the two tumors under analysis. The following proposition (see “Appendix F”) indicates when the expected payoff of an *A*-cell in ESS is strictly larger in the 2-type population game than in the 3-type population game.

#### Proposition 3

Let $$y_{A}$$ be a proportion of A-cells and let $$ \Gamma _{2}(v,c,y_{A})$$ and $$\Gamma _{3}(v,c,y_{A},x_{E})$$ with $$y_{A}<\bar{x }_{A}$$ and $$x_{E}<{\bar{x}}_{E}$$. Then $$U_{A}^{**}(y_{A})>U_{A}^{***}(y_{A},x_{E})$$.

**Fig. 3:**
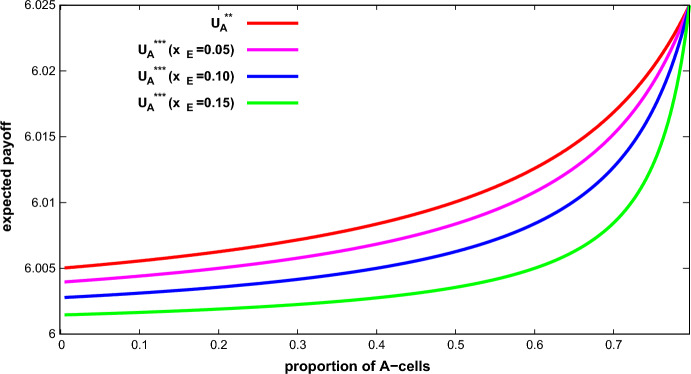
Expected payoff of the best-off *A*-cells in the ESS in a 2-type population game ($$U_{A}^{**}$$) and 3-type population game ($$ U_{A}^{***}$$ for different values of $$x_{E}$$) as functions of $$ x_{A}$$. Parameters: $$v=10$$, $$c=50$$, $$n=201$$ (Color figure online)

This result is illustrated in Fig. 3. Recall that the ESS for $x_{A}<{\bar{x}}_{A}$ in $\Gamma _{2}(v,c,x_{A})$ is $\left( 0,\tfrac{n-1}{ n-nx_{A}-1}\tfrac{v}{c}\right)$ while the ESS in $\Gamma _{3}(v,c,x_{A},x_{E})$ is $\left( 0,\frac{(n-1)v/c-nx_{E}}{n-nx_{A}-nx_{E}-1},1\right)$. In both games the best-off *A*-cells receive no aggression, while *E*-cells receive full aggression in the 3-type population game. *B* -cells receive some aggression in both games. The aggression received by *B* -cells in the 2-type population game is greater than in the 3-type population game. This difference confers an advantage to *A*-cells in the former.

## Discussion

Below, we discuss the consistency of our results obtained from extending the classic hawk–dove game to a heterogeneous population as outlined in Sect. 3 with the observations in the histological study on CCRCC described in Sect. 2.

To bridge the results from Sects. 2 and 3, recall that following the empirical oncology study in Manini et al. (2022), ITH is considered as low when the tumor has two different cell populations (grades) measured by histological parameters. Such tumors are represented in our model by 2-type population games. When the tumor has three or more identifiable cell populations, ITH is considered to be high. This case is represented by 3-type population games. In Table 1 the proportion of the highest grade can be interpreted as the proportion of the best-off type, that is, $$ x_{A}$$.

Proposition 1 says that in a 3-type population game an ESS exists when the proportions of the best-off and worst-off cells do not exceed certain thresholds. Beyond those thresholds, for the best-off and worst-off cells, the ESS has two types of cells that receive exactly the same aggression and therefore have the same expected payoff (see Proposition 2). In accordance with these results in the case study we do not expect to find patients with tumors that show high ITH and a large proportion of the highest grade cells. This is indeed the case and is reflected in Table 1. Indeed, high ITH is linked to proportions of $x_{A}\le 20\%$. From a genomic point of view, this result could be interpreted as a branching-type tumor becoming punctuated. Such an evolution has in fact been detected in several types of malignant tumors (Bao et al. 2021).

Proposition 3 says that the best-off cell type obtains a larger expected payoff in 2-type population games than in 3-type population games. This means that if two tumors with the same proportion of the highest grade are compared, the one with the higher ITH should be less aggressive than the one with the lower ITH. In other words, for the same proportion of the highest grade a three-cell-type tumor pursues a less aggressive course than a two-cell-type one. This result is also consistent with the findings in the case-study: Patients with cancer showing high ITH seem to have a better prognosis than those with cancers showing low ITH.

Figures 1 and  3 can be interpreted as follows: the higher the proportion of the highest grade cells, the more aggressive a tumor is (and the worse the prognosis is). Moreover, the rate is increasing. This means that the larger the proportion is, the larger the effect on the worsening of the prognosis is. More detailed data would be useful to check this conjecture.

At a practical level, our results suggest the idea that it is appropriate to preserve high ITH in tumors as a promising therapeutic strategy.Thus, a therapy using the tumor containment strategy instead of the conventional of maximum tolerable dose could be more effective since the former procedure forces tumor cells to divide its energy expenditure in two different tasks to survive, i.e. maintaining the tumor growth on one hand, and developing resistances to therapy on the other. Since the total amount of energy into the cell is limited by definition, both tasks will slow down allowing longer cancer survival rates in patients [24]. Moreover, the maximum tolerable dose has already been questioned using a game theory approach (Archetti 2021).

The extension of the classic game of hawk and dove to heterogeneous populations seems to support the results presented in the case study. We believe that our contribution opens up a path for future research into developing case studies where low and high ITH in other types of cancer are analyzed in a way that enables the robustness of the hawk–dove game model to be confirmed for heterogeneous populations.

From a clinical point of view, the results highlight the importance of therapies focused on maintaining high levels of intra-tumor heterogeneity, with a greater diversity of cancer cells, in order to try to slow down the progress of cancer and decrease its clinical aggressiveness.

## Data Availability

All data generated or analyzed during this study are included in this published article [and its supplementary information files].

## Appendix A

In a 2-type population game the expected payoff of *A*-cells in the ESS increases with $x_{A}$ for $x_{A}<{\bar{x}}_{A}$ and decreases for $x_{A}>\bar{ x}_{A}$.

### Proof

From (2), compute:

$$\begin{aligned} \frac{d}{dx_{A}}\left[ \frac{v}{2}\left( 1-\frac{v}{c}\right) +\frac{v^{2}}{ 2c}\tfrac{2n(1-x_{A})-1}{n-nx_{A}-1}\right]= & {} \frac{v^{2}n}{2c\left( n-nx_{A}-1\right) ^{2}}>0 \\ \frac{d}{dx_{A}}\left[ \frac{v}{2}\left( 1-\frac{v}{c}\right) +\frac{v}{2c} \frac{v(n-1)+cn(1-x_{A})}{n-1}\right]= & {} -\frac{vn}{2(n-1)}<0 \\ \frac{d}{dx_{A}}\left[ \frac{v}{2}\left( 1-\frac{v}{c}\right) +\frac{c-v}{c} \frac{vn(1-x_{A})}{nx_{A}-1}\right]= & {} -\frac{c-v}{c}\frac{vn(n-1)}{\left( nx_{A}-1\right) ^{2}}<0. \end{aligned}$$

Therefore, $$\frac{d}{dx_{A}}\left[ U_{A}^{**}(x_{A})\right] >0$$ if $$ x_{A}<{\bar{x}}_{A}$$ and $$\frac{d}{dx_{A}}\left[ U_{A}^{**}(x_{A}) \right] <0$$ if $$x_{A}>{\bar{x}}_{A}$$. $$\square $$

## Appendix B

Whenever cells choose the same strategy, i.e. $$(\alpha _{A},\alpha _{B},\alpha _{E})=(\beta _{A},\beta _{B},\beta _{E})$$, the following holds: $$ U_{I}>U_{J}$$ is equivalent to $$\alpha _{I}<\alpha _{J}$$ (for $$I,J\in \{{ A,B,E\}};\ I\ne J$$).

### Proof

For $$(\alpha _{A},\alpha _{B},\alpha _{E})=(\beta _{A},\beta _{B},\beta _{E}) $$, (3) becomes

$$\begin{aligned} U_{A}= & {} \frac{nx_{A}-1}{n-1}u(\alpha _{A},\alpha _{A})+\frac{ n(1-x_{A}-x_{E})}{n-1}u(\alpha _{B},\alpha _{A})+\frac{nx_{E}}{n-1}u(\alpha _{E},\alpha _{A}) \\ U_{B}= & {} \frac{nx_{A}}{n-1}u(\alpha _{A},\alpha _{A})+\frac{ n(1-x_{A}-x_{E})-1}{n-1}u(\alpha _{B},\alpha _{A})+\frac{nx_{E}}{n-1} u(\alpha _{E},\alpha _{A}) \\ U_{E}= & {} \frac{nx_{A}}{n-1}u(\alpha _{A},\alpha _{A})+\frac{n(1-x_{A}-x_{E}) }{n-1}u(\alpha _{B},\alpha _{A})+\frac{nx_{E}-1}{n-1}u(\alpha _{E},\alpha _{A}) \end{aligned}$$

Plugging (1) into the above equation gives

$$\begin{aligned}{} & {} U_{A} =\frac{v}{2}(1-\alpha _{A})+\frac{c}{2(n-1)}(\frac{v}{c}-\alpha _{A})\\{} & {} \quad \left[ nx_{A}\alpha _{A}+n(1-x_{A}-x_{E})\alpha _{B}+nx_{E}\alpha _{E}\right] -\frac{c}{2(n-1)}(\frac{v}{c}-\alpha _{A})\alpha _{A} \\{} & {} U_{B} =\frac{v}{2}(1-\alpha _{B})+\frac{c}{2(n-1)}(\frac{v}{c}-\alpha _{B})\\{} & {} \quad \left[ nx_{A}\alpha _{A}+n(1-x_{A}-x_{E})\alpha _{B}+nx_{E}\alpha _{E}\right] -\frac{c}{2(n-1)}(\frac{v}{c}-\alpha _{B})\alpha _{B} \\{} & {} U_{E} =\frac{v}{2}(1-\alpha _{E})+\frac{c}{2(n-1)}(\frac{v}{c}-\alpha _{E})\\{} & {} \quad \left[ nx_{A}\alpha _{A}+n(1-x_{A}-x_{E})\alpha _{B}+nx_{E}\alpha _{E}\right] -\frac{c}{2(n-1)}(\frac{v}{c}-\alpha _{E})\alpha _{E} \end{aligned}$$

The difference between two pairs of equations gives

8 $$\begin{aligned}{} & {} U_{A}-U_{B} = \frac{nv}{2(n-1)}(\alpha _{B}-\alpha _{A})+\frac{c}{2(n-1)} \left( \alpha _{B}-\alpha _{A}\right) \nonumber \\{} & {} \quad \left[ nx_{A}\alpha _{A}+n(1-x_{A}-x_{E})\alpha _{B}+nx_{E}\alpha _{E}\right] -\frac{c}{2(n-1)}\left( \alpha _{B}^{2}-\alpha _{A}^{2}\right) \nonumber \\{} & {} U_{A}-U_{B} \nonumber \\{} & {} \quad =\frac{\alpha _{B}-\alpha _{A}}{2(n-1)}\left[ nv+cnx_{A}\alpha _{A}+cn(1-x_{A}-x_{E})\alpha _{B}+cnx_{E}\alpha _{E}-c\left( \alpha _{B}+\alpha _{A}\right) \right] \nonumber \\{} & {} U_{A}-U_{B} \nonumber \\{} & {} =\frac{\alpha _{B}-\alpha _{A}}{2(n-1)}\left[ nv+c\left( nx_{A}-1\right) \alpha _{A}+c\left( n-nx_{A}-nx_{E}-1\right) \alpha _{B}+cnx_{E}\alpha _{E}\right] \end{aligned}$$

Given that there is at least one cell of each type, we have $x_{A}>\frac{1}{n }$, $1-x_{A}-x_{E}>\frac{1}{n}.$ Thus $U_{A}>U_{B}$ is equivalent to $\alpha _{A}<\alpha _{B}$. Following the same reasoning we have the equivalence between $U_{B}>U_{E}$ and $\alpha _{B}<\alpha _{E}$. Thus, $U_{A}>U_{B}>U_{E}$ iff $\alpha _{A}<\alpha _{B}<\alpha _{E}$.$\square$

## Appendix C

In a 3-type population game strategy $\left( 0,\frac{(n-1)v/c-nx_{E}}{ n-nx_{A}-nx_{E}-1},1\right)$ is an ESS if $x_{E}<{\bar{x}}_E$ and $x_{A}<\bar{ x}_A$.

### Proof

First, check that strategy $\left( 0,\frac{(n-1)v/c-nx_{E}}{n-nx_{A}-nx_{E}-1 },1\right)$ is a best response to itself.

From (5), the optimal choice of a cell if the opponent plays $(\beta _{A},\beta _{B},\beta _{E})$ is to play $\alpha _{I}=1$ whenever $f_{I}(\beta _{A},\beta _{B},\beta _{E})>0$, $\alpha _{I}=0$ whenever $f_{I}(\beta _{A},\beta _{B},\beta _{E})<0$ and $\alpha _{I}\in [0,1]$ whenever $f_{I}(\beta _{A},\beta _{B},\beta _{E})=0$ where $I=A,B,E$. If the opponent plays $\left( 0,\beta _{B},1\right)$ the optimal choice of a cell is $\alpha _{A}=0$, $\alpha _{B}=\beta _{B}$ (with $0\le \alpha _{B}\le 1$ ) and $\alpha _{E}=1$ as long as $f_{A}\left( 0,\beta _{B},1\right) <0$, $f_{B}\left( 0,\beta _{B},1\right) =0$ and $f_{E}\left( 0,\beta _{B},1\right) >0$.

Equality $f_{B}\left( 0,\beta _{B},1\right) =0$ is satisfied if $\frac{v}{ c} \left( 1-\frac{1}{n}\right) -(1-x_A-x_E)\beta _B-x_E+\frac{\beta _B}{n}=0$ or equivalently $\frac{v}{c}\left( n-1\right) -(n-nx_A-nx_E)\beta _B-nx_E+ \beta _B=0$, leading to $\beta _{B}=\frac{(n-1)v/c-nx_{E}}{n-nx_{A}-nx_{E}-1}$.

Condition $\beta _{B}>0$ gives $(n-1)v/c-nx_{E}$ $>0$ or equivalently $x_{E}< {\bar{x}}_E$ (assuming there is strictly more than one cell of each type, i.e., $nx_{B}=n-nx_{A}-nx_{E}>1$).

Condition $\beta _{B}<1$ gives $(n-1)v/c-nx_{E}<n-nx_{A}-nx_{E}-1$, or $x_{A}<{\bar{x}}_{A}$. It remains to be confirmed that $f_{A}\left( 0,\frac{ (n-1)v/c-nx_{E}}{n-nx_{A}-nx_{E}-1},1\right) <0$ and $f_{E}\left( 0,\frac{ (n-1)v/c-nx_{E}}{n-nx_{A}-nx_{E}-1},1\right) >0$ hold at $\left( 0,\frac{ (n-1)v/c-nx_{E}}{n-nx_{A}-nx_{E}-1},1\right)$, which with simple algebraic manipulations can be proved to be true.

Second, it remains to be confirmed that strategy $\left( 0,\frac{(n-1)v/c-nx_{E}}{ n-nx_{A}-nx_{E}-1},1\right)$ which is a best response to $\left( 0,\frac{ (n-1)v/c-nx_{E}}{n-nx_{A}-nx_{E}-1},1\right)$ cannot be invaded by a mutant strategy.

Let the opponent play $(\gamma _{A},\gamma _{B},\gamma _{E})$, as a best response to $\left( 0,\frac{(n-1)v/c-nx_{E}}{n-nx_{A}-nx_{E}-1},1\right)$. It must be checked that the expected payoff obtained when playing $\left( 0, \frac{(n-1)v/c-nx_{E}}{n-nx_{A}-nx_{E}-1},1\right)$ is larger than when playing $(\gamma _{A},\gamma _{B},\gamma _{E})$. As checked above $f_{A}\left( 0,\frac{(n-1)v/c-nx_{E}}{n-nx_{A}-nx_{E}-1},1\right) <0$, $f_{B}\left( 0,\frac{(n-1)v/c-nx_{E}}{n-nx_{A}-nx_{E}-1},1\right) =0$ and $f_{E}\left( 0,\frac{(n-1)v/c-nx_{E}}{n-nx_{A}-nx_{E}-1},1\right) >0$. Thus, $\gamma _{A}=0$, $0<\gamma _{B}<1$, and $\gamma _{E}=1$. From (5) we write the difference in expected payoffs when playing $\left( 0,\frac{ (n-1)v/c-nx_{E}}{n-nx_{A}-nx_{E}-1},1\right)$ and $\left( 0,\gamma _{B},1\right)$ whenever the opponent plays $\left( 0,\gamma _{B},1\right)$:

$$\begin{aligned}{} & {} f\left( 0,\gamma _{B},1\right) +f_{A}\left( 0,\gamma _{B},1\right) \cdot 0+f_{B}\left( 0,\gamma _{B},1\right) \text { }\\{} & {} \quad \frac{(n-1)v/c-nx_{E}}{ n-nx_{A}-nx_{E}-1}+f_{E}\left( 0,\gamma _{B},1\right) \cdot 1 \\{} & {} \quad -f\left( 0,\gamma _{B},1\right) -f_{A}\left( 0,\gamma _{B},1\right) \cdot 0-f_{B}\left( 0,\gamma _{B},1\right) \gamma _{B}-f_{E}\left( 0,\gamma _{B},1\right) \cdot 1 \\{} & {} \quad =f_{B}\left( 0,\gamma _{B},1\right) \left( \frac{(n-1)v/c-nx_{E}}{ n-nx_{A}-nx_{E}-1}-\gamma _{B}\right) \\{} & {} \quad =\frac{c}{2}(1-x_{A}-x_{E})\\{} & {} \quad \left( \frac{v}{c}-\frac{nx_{E}}{n-1}-\frac{ n-nx_{A}-nx_{E}-1}{n-1}\gamma _{B}\right) \left( \frac{(n-1)v/c-nx_{E}}{ n-nx_{A}-nx_{E}-1}-\gamma _{B}\right) \\{} & {} \quad =\frac{c}{2}(1-x_{A}-x_{E})\frac{n-nx_{A}-nx_{E}-1}{n-1}\left( \frac{ (n-1)v/c-nx_{E}}{n-nx_{A}-nx_{E}-1}-\gamma _{B}\right) ^{2} \\{} & {} \quad >0\,\text { if }\,\gamma _{B}\ne \frac{(n-1)v/c-nx_{E}}{n-nx_{A}-nx_{E}-1} \end{aligned}$$

Thus, the expected payoff is strictly larger when playing $\left( 0,\frac{ (n-1)v/c-nx_{E}}{n-nx_{A}-nx_{E}-1},1\right)$ than $\left( 0,\gamma _{B},1\right)$ and strategy $\left( 0,\frac{(n-1)v/c-nx_{E}}{ n-nx_{A}-nx_{E}-1},1\right)$ is an ESS. $\square$

## Appendix D

In a 3-type population game, the aggression suffered by *B*-cells in the ESS increases with the proportion of *A*-cells, $x_{A}$; but it decreases with the proportion of *E*-cells, $x_{E}$ for $x_{A}<{\bar{x}}_{A}$ and $x_{E}<\bar{ x}_{E}.$

### Proof

From (6), compute:

$$\begin{aligned} \frac{\partial }{\partial x_{A}}\left[ \frac{(n-1)v/c-nx_{E}}{ n-nx_{A}-nx_{E}-1}\right] =\frac{n\left( (n-1)v/c-nx_{E}\right) }{\left( n-nx_{A}-nx_{E}-1\right) ^{2}}>0 \end{aligned}$$

given that $x_{E}<(1-\frac{1}{n})\frac{v}{c}={\bar{x}}_{E}$ in the ESS.

$$\begin{aligned} \frac{\partial }{\partial x_{E}}\left[ \frac{(n-1)v/c-nx_{E}}{ n-nx_{A}-nx_{E}-1}\right] =\frac{-n\left( n-nx_{A}-1\right) +n\left( (n-1)v/c\right) }{\left( n-nx_{A}-nx_{E}-1\right) ^{2}}<0 \end{aligned}$$

given that $x_{A}<(1-\frac{1}{n})(1-\frac{v}{c})={\bar{x}}_{A}$ in the ESS. $\square$

## Appendix E

In the 3-type population game the expected payoff of each *A*-cell in the ESS increases with $x_{A}$ and decreases with $x_{E}$.

### Proof

From (7), compute:

$$\begin{aligned}{} & {} \frac{\partial }{\partial x_{A}}\left[ U_{A}^{***}(x_{A},x_{E}) \right] \\{} & {} \quad =\frac{\partial }{\partial x_{A}}\left[ \frac{v}{2}\left( 1-\frac{v }{c}\right) +\frac{v^{2}}{2c}\frac{2n-2nx_{A}-2nx_{E}-1}{n-nx_{A}-nx_{E}-1}- \frac{v}{2}\frac{nx_{E}}{\left( n-nx_{A}-nx_{E}-1\right) \left( n-1\right) } \right] \\{} & {} \quad =\frac{v}{2}\left[ \frac{v}{c}\frac{(n-1)}{n}-x_{E}\right] \frac{n}{\left( n-nx_{A}-nx_{E}-1\right) ^{2}}\frac{n}{n-1}>0. \end{aligned}$$

given that $x_{E}<(1-\frac{1}{n})\frac{v}{c}={\bar{x}}_{E}$ in the ESS.

$$\begin{aligned}{} & {} \frac{\partial }{\partial x_{E}}\left[ U_{A}^{***}(x_{A},x_{E}) \right] \\{} & {} \quad =\frac{\partial }{\partial x_{E}}\left[ \frac{v}{2}\left( 1-\frac{v }{c}\right) +\frac{v^{2}}{2c}\frac{2n-2nx_{A}-2nx_{E}-1}{n-nx_{A}-nx_{E}-1}- \frac{v}{2}\frac{nx_{E}}{\left( n-nx_{A}-nx_{E}-1\right) \left( n-1\right) } \right] \\{} & {} \quad =\frac{v}{2}\left[ \frac{v}{c}\frac{(n-1)}{n}-\frac{(n-1)}{n}+x_{A}\right] \frac{n}{\left( n-nx_{A}-nx_{E}-1\right) ^{2}}\frac{n}{n-1} \\{} & {} \quad =\frac{v}{2}\left[ x_{A}-\left( 1-\frac{v}{c}\right) \left( 1-\frac{1}{n} \right) \right] \frac{n}{\left( n-nx_{A}-nx_{E}-1\right) ^{2}}\frac{n}{n-1} <0. \end{aligned}$$

given that $x_{A}<(1-\frac{1}{n})(1-\frac{v}{c})={\bar{x}}_{A}$ in the ESS.

Therefore, we have $$\frac{\partial }{\partial x_{A}}\left[ U_{A}^{***}(x_{A},x_{E}) \right] >0$$ and $$\frac{\partial }{\partial x_{E}}\left[ U_{A}^{***}(x_{A},x_{E}) \right] <0$$. $$\square $$

## Appendix F

For $$x_{A}<{\bar{x}}_{A}$$ and $$x_{E}<{\bar{x}}_{E}$$ the following holds: $$ U_{A}^{**}(x_{A})>U_{A}^{***}(x_{A},x_{E})$$.

### Proof

From (2) and (7), compute:

$$\begin{aligned}{} & {} U_{A}^{**}(x_{A})-U_{A}^{***}(x_{A},x_{E}) =\frac{v^{2}}{ 2c} \left( \frac{2n-2nx_{A}-1}{n-nx_{A}-1}-\frac{2n-2nx_{A}-2nx_{E}-1}{ n-nx_{A}-nx_{E}-1}\right) \\{} & {} \quad +\frac{v}{2}\frac{nx_{E}}{\left( n-nx_{A}-nx_{E}-1\right) \left( n-1\right) }. \end{aligned}$$

First, note that:

$$\begin{aligned}{} & {} \frac{v^{2}}{2c}\left( \frac{2n-2nx_{A}-1}{n-nx_{A}-1}-\frac{ 2n-2nx_{A}-2nx_{E}-1}{n-nx_{A}-nx_{E}-1}\right) \\{} & {} \quad =\frac{v^{2}}{2c}\frac{(2n-2nx_{A}-1)(-nx_{E})+2nx_{E}(n-nx_{A}-1)}{(n-nx_{A}-1)(n-nx_{A}-nx_{E}-1)}\\{} & {} \quad =\frac{v^{2}}{2c}\frac{-nx_{E}}{(n-nx_{A}-1)(n-nx_{A}-nx_{E}-1)}. \end{aligned}$$

This gives:

$$\begin{aligned}{} & {} U_{A}^{**}(x_{A},x_{E})-U_{A}^{***}(x_{A},x_{E})\\{} & {} \quad =\frac{v^{2}}{2c}\frac{-nx_{E}}{(n-nx_{A}-1)(n-nx_{A}-nx_{E}-1)}+\frac{v}{2} \frac{nx_{E}}{\left( n-nx_{A}-nx_{E}-1\right) \left( n-1\right) } \\{} & {} \quad =\frac{v}{2}\frac{nx_{E}}{\left( n-nx_{A}-nx_{E}-1\right) \left( n-1\right) (n-nx_{A}-1)}\left[ -\frac{v}{c}(n-1)+(n-nx_{A}-1)\right] \\{} & {} \quad =\frac{v}{2}\frac{nx_{E}}{\left( n-nx_{A}-nx_{E}-1\right) \left( n-1\right) (n-nx_{A}-1)}\left[ (n-1)\left( 1-\frac{v}{c}\right) -nx_{A}\right] \\{} & {} \quad >0\text { given that }x_{A}<\left( 1-\frac{1}{n}\right) \left( 1-\frac{v}{c} \right) ={\bar{x}}_A. \end{aligned}$$

Thus, for $$x_{A}<{\bar{x}}_A$$ and $$x_{E}<{\bar{x}}_E$$, we have $$ U_{A}^{**}(x_{A})>U_{A}^{***}(x_{A},x_{E})$$. $$\square $$

## Footnotes

1 Note that the ESS corresponds to the outcome of a replicator dynamics system when the latter reaches an asymptotically stable state.

2 See the discussion in Beckman et al. (2020),

3 The complete table is given in Manini et al. (2022). Here we omit data such as gender, age, number of samples per case, number of months of follow-up and highest grade encountered. Note that we have maintained the numbering of the original table.

4 This assumption contrasts with the traditional assumption in game theory (usually players know their own type but not that of their opponent).

5 That is, in one ESS, *A*-cells face less aggression and have a larger payoff than *B*-cells. In the other ESS the opposite holds.
